# Osteopontin: A Versatile Biomarker—Insights and Innovations from Three Decades of Research

**DOI:** 10.3390/biomedicines12081647

**Published:** 2024-07-24

**Authors:** Hugo Abreu, Giuseppe Cappellano

**Affiliations:** 1 Department of Health Sciences, Interdisciplinary Research Center of Autoimmune Diseases-IRCAD, Università del Piemonte Orientale, 28100 Novara, Italy; hugo.abreu@uniupo.it; 2 Center for Translational Research on Autoimmune and Allergic Diseases-CAAD, Università del Piemonte Orientale, 28100 Novara, Italy

This second Biomedicines Special Issue—“30 Years of osteopontin (OPN) Milestones and Future Avenues 2.0”—follows the first one [1] in this series by further exploring the importance of OPN in the pathogenesis of several diseases [2]; its potential as a diagnostic and prognostic biomarker is also explored [3]. This collection encompasses twelve papers investigating OPN’s various mechanisms of action in pathological conditions, highlight its potential role as a prognostic biomarker for Hodgkin’s lymphoma and its predictive value for acute pancreatitis disease progression. Additionally, OPN is explored as a therapeutic target for treating chronic subdural hematoma and melanoma.

Firstly, De Re et al. linked OPN with a poorer prognosis in classical Hodgkin’s lymphoma patients who underwent the same treatment. In particular, this was manifested with an increase in tissue necrosis and inflammation. The authors also report a positive correlation between positron emission tomography scans and OPN expression in tumor tissue sections prior to therapy; although, its levels could not improve the prediction and prognosis assessment of the technique. Lastly, the overexpression of OPN was associated with a dysregulation in several chemokines and cytokines, but also with dendritic cells’ infiltration; this might help elucidate possible pathological mechanisms and develop prognostic strategies for Hodgkin’s lymphoma [4].

On the other hand, Chunder et al. focused on the impact of OPN on B-cells, in the context of multiple sclerosis (MS). Although OPN is present in autopsied brain tissue samples of patients without B-cell infiltration, it is highly expressed in B-cell aggregates. This was confirmed in vitro, since B-cells treated with recombinant OPN show an increased number of aggregates compared to the untreated control. Conversely, OPN led to the downregulation of CD80 and CD86, two co-stimulatory molecules involved in B-cell activation, together with a lower secretion of inteleukin-6, indicating the dual role of OPN in regulating B-cell response [5].

Aiming to find a reliable biomarker for acute pancreatitis, Wirestam et al. postulated that circulating OPN (cOPN) could be an interesting research target. The authors detected, at the time of admission, higher cOPN expression in patient plasma samples compared to controls; however, this did not allow for the discrimination of the severity of the disease. Still, patients with a more severe manifestation of the disease show an increase in cOPN expression throughout the following three days, indicating that measuring its levels over time could predict disease progression [6].

Similarly, Saup et al. investigated the role of cOPN; the authors used a mouse melanoma model, in which OPN was overexpressed through adeno-associated virus-mediated transduction. This led to an increased expression of several genes involved in tumor development in the lungs, a common metastatic site for melanoma cells. Although the significant increase in cOPN without altering local tissue expression stimulated tumor growth, it did not affect the level of metastasis, which shows that OPN could exert diverse effects throughout melanoma progression [7], possibly by binding to its receptors, as previously described by Raineri et al. [8].

Osuka et al. evaluated the presence and expression of the N-terminal half of OPN (OPN-N), which is formed naturally via cleavage through thrombin action, in chronic subdural hematoma (CSDH) fluid. Thrombin cleavage of the full-length secreted OPN (OPN-FL) also generates a C-terminal (OPN-C) fragment, which, together with OPN-N exhibits distinct biological activities compared to OPN-FL. These activities include leukocyte chemotaxis and adhesion, as well as promoting cell survival and differentiation. It is hypothesized that this gain of function following cleavage is a result of the exposure of regions of the OPN protein that are not accessible in the OPN-full length molecule [9]. In the case of the work developed by Osuka and colleagues, OPN-N levels in CSDH fluid were found to be significantly higher than those in the serum. Using mouse endothelial cells in vitro, the authors also demonstrated that CSDH fluid is able to phosphorylate FAK and ERK, which are downstream components of the angiogenic signaling pathways present in the outer membrane of CSDHs; this is possibly carried out through the binding of OPN-N to integrins α9 and β1. With this, targeting OPN-N and/or the integrins α9 and β1 pathway could represent a valuable strategy for CSDH treatment [10]. It would be of interest to investigate the effects of the OPN-C fragment, considering that the in vivo neutralization of each fragment exerts opposite roles in a mouse model of MS [11].

This Special Issue also contains a myriad of literature reviews, evidencing the current knowledge on OPN, its variances, binding partners, and role in both physiological and pathological contexts. Panda et al. highlighted the impact of OPN in cancer-related processes, based on its splice variants and its binding to several interactors [12]. On the other hand, Hao et al. focused on OPN-based mechanisms that confer resistance to several drugs and radiation doses, such as drug transport, apoptosis, stemness, energy metabolism, and autophagy; the authors defended the fact that targeting OPN as a form of cancer therapy can improve the treatment outcome [13].

A deeper look into the tissue-specific effects of OPN reveal its important role in pathologies related to different organs. Silver and Popovics showed how OPN influences prostate hyperplasia, cancer, and inflammation, focusing on the differential secretion of this molecule by different cell types including foam cells, epithelial cells, and stromal cells [14]. Given the presence of OPN in atherosclerotic plaque, Kadoglou et al. discussed its role in atherosclerotic cardiovascular diseases, in which it might act as an inflammation and calcification mediator. In fact, the authors present valuable insights on the current clinical trials linking OPN with coronary artery disease and calcification, revealing this molecule as a possible prognostic biomarker [15]. In the context of pulmonary hypertension and ventricular failure, Mamazhakypov et al. exposed OPN’s known mechanisms of action in pulmonary vascular remodeling through its specific effects on the different cell types present; this was validated using several animal models [16]. Not only that, OPN is also involved in amyloid-β and tau clearance, whose accumulation in the brain is a main feature of Alzheimer’s disease. Thus, Lalwani et al. investigated how OPN can influence disease pathology and progression, showing the contrasting effects of this molecule regarding the promotion of inflammation (i.e., tissue damage, the Yin), but also its contribution to regeneration/neuroprotection (the Yang) [17,18], which is triggered by inflammatory responses. Lastly, Sinha et al. suggested that OPN could be a valid biomarker for various forms of chronic kidney disease. Particularly, OPN-N has been closely linked to renal inflammation and fibrosis, suggesting that targeting this variant could represent a potential therapy for some kidney-associated pathologies [19].

Overall, the findings exhibited within the scope of this Special Issue highlight the variety of effects exerted by OPN, influencing many biological pathways and therefore resulting in several altered pathologies. Given its pleiotropic role, continued research on OPN as a diagnostic, prognostic, and therapeutic biomarker is crucial in the process of deepening our understanding of the mechanisms involved in the pathogenesis of several types of cancer, as well as autoimmune and inflammatory diseases. By elucidating these mechanisms, we can enhance our ability to diagnose these diseases more accurately, predict their progression, and develop targeted therapies that improve patient outcomes.
